# Association between Sleep Duration and Hypertension among Adults in Southwest China

**DOI:** 10.5334/gh.1100

**Published:** 2022-02-21

**Authors:** Jie He, Quan He

**Affiliations:** 1 The First Affiliated Hospital of Chongqing Medical University, CN

**Keywords:** Short sleep duration, Long sleep duration, Hypertension

## Abstract

**Objective:**

This study aimed to evaluate the association between sleep duration and hypertension among adults in southwest China.

**Methods:**

Baseline variables were collected from a representative sample of 20,053 adults aged 23–98 years in southwest China who received physical examinations from January 2019 to December 2020. All participants were categorized into either a hypertension group or a non-hypertension group. Sleep duration was classified as short (<6 h/day), normal (6–8 h/day),or long (>8 h/day). Baseline variables were compared between individuals with and without hypertension by rank-sum tests for two independent samples or χ^2^ tests for nonparametric data. Multivariate logistic regression analysis was performed to evaluate the association between sleep duration and hypertension.

**Results:**

The overall incidence of hypertension was 51.2%. Unadjusted analysis showed that the risk of hypertension was higher in individuals with short (<6h/day) or long (>8h/day) sleep durations compared with those with a normal (6–8 h/day) sleep duration. The risk of hypertension was significantly increased by 30.1% in participants with a long (>8h/day) sleep duration compared with those with a normal (6–8h/day) sleep duration (OR = 1.301, *P* < 0.010, 95%CI = 1.149–1.475). The risk of hypertension was also increased by 1.1% in participants with a short (<6h/day) sleep duration compared with participants with a normal (6–8h/day) sleep duration, but the difference was not significant (OR = 1.011, *P* = 0.849, 95%CI = 0.905–1.129). After fully adjusting for confounding factors (model 4), the risk of hypertension was increased significantly (by 25%) in individuals with a short (<6h/day) sleep duration (OR = 1.25, *P* = 0.02, 95%CI = 1.036–1.508) but not in those with a long (>8h/day) sleep duration (17.5% increase) compared with participants with a normal (6–8h/day) sleep duration (OR = 1.175, *P* = 0.144, 95%CI = 0.946–1.460).

**Conclusion:**

The results of this study indicate that a short (<6h/day) sleep duration is related to an increased risk of hypertension, suggesting that sleep helps to protect against hypertension.

## Introduction

Hypertension is a leading cause of cardiovascular death, stroke, kidney failure, and disability. The early detection, appropriate treatment, and better control of hypertension have significant health and economic benefits. Current actions considered to reduce the risk of hypertension include: maintaining a healthy body weight (BMI = 18.5–24.9 kg/m^2^) and waist circumference (102 cm for men and 88 cm for women); addressing behavioral risk factors such as an unhealthy diet, harmful use of alcohol, lack of exercise, and smoking; following a diet including more fruit and vegetables, and low-fat dairy products; and limiting sodium consumption from all sources.

Sleep is a normal part of life, but an increasing number of people have insufficient sleep, which can seriously affect their health. Numerous studies have demonstrated that sleep is closely related to hypertension, with both short and long sleep duration associated with a series of metabolic disorders [4], such as abnormal blood glucose and lipids, impaired vascular endothelial function, and obesity. However, there have been clear discrepancies among the findings of these studies. Certain studies found that both short and long sleep durations were associated with an increased risk of hypertension [1,2,13,14], while several studies only identified a short sleep duration as associated with an increased risk of hypertension [3,6,7,8,12,15], and yet other studies denied this association [5,11,17]. The objective of the current study was to evaluate the association between self-reported sleep duration and hypertension among adults in southwest China.

## Subjects and Methods

### Subjects

**Figure 1:**
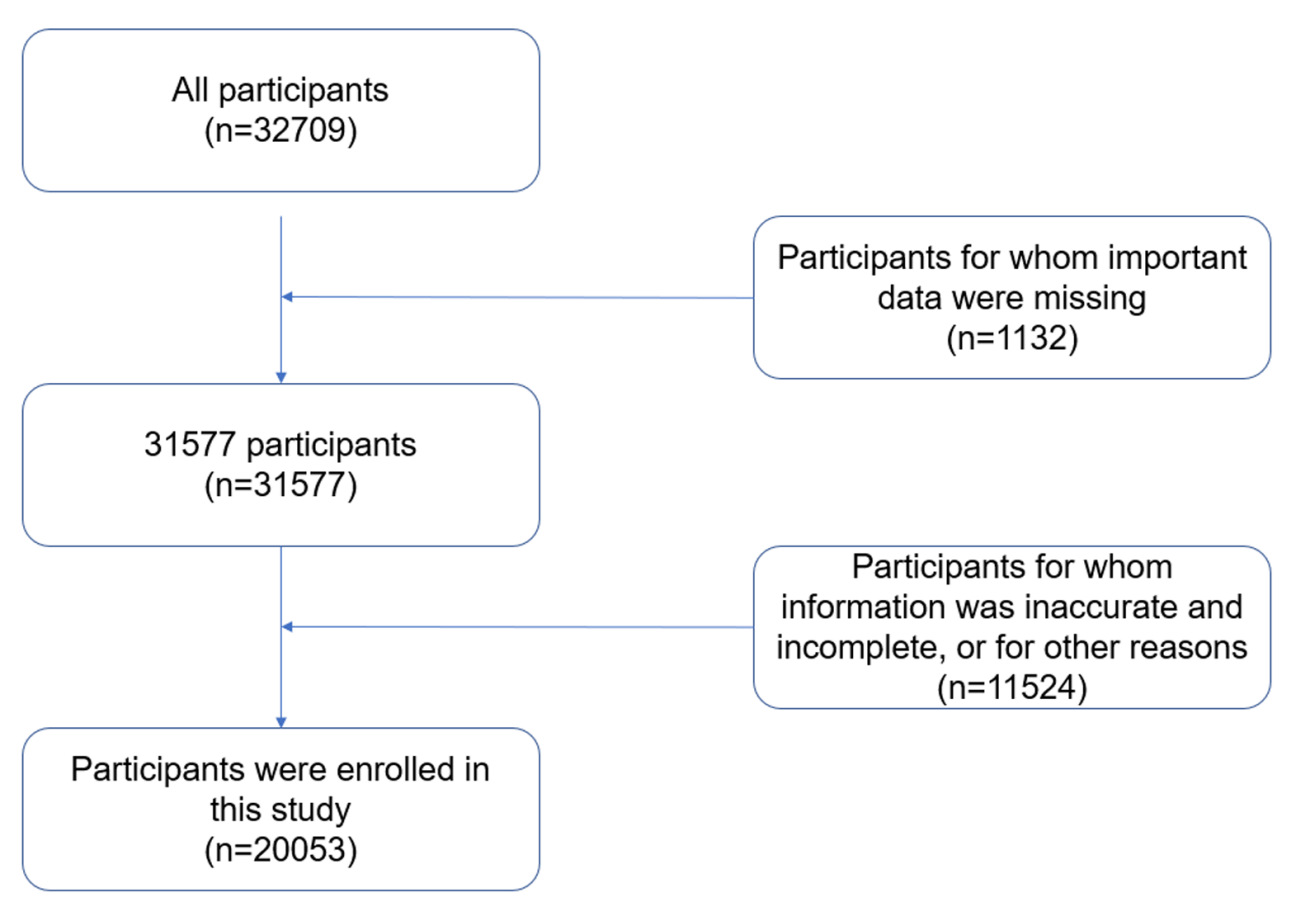
Flowchart of study participants.

We conducted a cross-sectional study to evaluate the association between sleep duration and hypertension in a multi-stage stratified random sample of 32,709 adults who participated in community medical examinations in southwest China from August 2018 to December 2020. We excluded 1,132 participants for whom important data were missing and 11,524 participants for whom information was inaccurate and incomplete, or for other reasons. Finally, 20,053 participants were included in this study (***Figure 1***). Participants with a systolic blood pressure ≥140 mmHg and/or a diastolic blood pressure ≥90 mmHg, or who were using antihypertensive medication were classified as the hypertension group, and the others were classified as the non-hypertension group. Subjects who met the inclusion criteria were informed of the research content and enrolled in the study after signing an informed consent form before the survey.

### Methods

#### Definition of hypertension

Participants were considered to have hypertension if they answered ‘yes’ to the question, ‘Have you ever been told by a doctor that you have hypertension, also known as high blood pressure?’, or if they self-reported antihypertensive drug use. Participants underwent blood pressure measurements three times with three minutes intervals on the right or left arm using an Omron electronic sphygmomanometer (which uses Intellisense® intelligent compression technology for accurate and convenient blood pressure measurement. Manufacturer: Omron (Dalian) Co., Ltd. Model: HBP-1300, and has been corrected) after being seated for at least 10 minutes. Systolic blood pressure (SBP) and diastolic blood pressure (DBP) were defined as the average of three readings, and SBP ≥ 140 mmHg or DBP ≥ 90 mmHg was also considered to indicate hypertension.

#### Assessment of sleep duration

Sleep duration was assessed using the question: ‘How much sleep do you usually get at night per day?”. Sleep duration was divided into three categories: short (<6 h/day), normal (6–8 h/day), and long sleep duration (>8 h/day), with normal sleep duration used as the reference group.

#### Other covariates

Heart rate (HR) was measured three times with 30-second intervals on the participants’ right or left arm using an Omron electronic sphygmomanometer (which uses Intellisense® intelligent compression technology for accurate and convenient blood pressure measurement. Manufacturer: Omron (Dalian) Co., Ltd. Model: HBP-1300, and has been corrected) after being seated for at least 10 minutes, and HR was defined as the average of the three readings. Body height and weight were measured using multifunctional electronic scales while the participant was wearing a thin T-shirt and no shoes. Body mass index (BMI) (kg/m^2^) was calculated by dividing the body weight in kilograms by the height square in meters. Blood glucose was measured using a single glucometer measurement in the distal finger before breakfast.

The following baseline population information was input: demographic information (e.g. sex, age, educational status, BMI), lifestyle (e.g. smoking, drinking, outdoor activities, vegetable consumption, fruit consumption), as well as disease history (e.g. hypertension, diabetes, hyperlipidemia, hyperhomocysteinemia, coronary vessel disease (CVD), chronic hepatic failure (CHF), chronic renal failure (CRF), atrial fibrillation (AF), cerebral infarction, cerebral hemorrhage, use of antihypertensives, use of lipid-lowering agents). All the variables are shown in ***Table 1***.

**Table 1:** The variable assignment.


VARIABLE	THE ASSIGNMENT

Sex	1 = male; 2 = female

Age	68 ± 10

BMI (kg/m^2^)	24 ± 3

HR (times/min)	74 ± 11

Blood glucose(mmol/L)	5.9 ± 1.9

Educational status	1 = primary, 2 = middle, 3 = high, 4 = college, 5 = master

Smoking	0 = never, 1 = current, 2 = past, 3 = don’t smoke but has a long history of secondhand smoke inhalation

Drinking	0 = never,1 = current, 2 = past

Outdoor activities (hours/week)	1 =≥ 20, 2 = 5–20, 3 =≤ 5

Vegetable consumption (days/week)	1 =≥ 5, 2 = 3–5, 3 =≤ 3

Fruit consumption (day/week)	1 =< 3, 2 =≥ 3

Sleep duration (hours/days)	1 = 6–8, 2 =< 6, 3 => 8

Hypertension	0 = no, 1 = yes

Diabetes	0 = no, 1 = yes

Hyperlipidemia	0 = no, 1 = yes

Hyperhomocysteinemia	0 = no, 1 = yes

CVD	0 = no, 1 = yes

CHF	0 = no, 1 = yes

CRF	0 = no, 1 = yes

AF	0 = no, 1 = yes

Cerebral infarction	0 = no, 1 = yes

Cerebral hemorrhage	0 = no, 1 = yes

Use of antihypertensives	0 = no, 1 = yes

Use of lipid-lowering agents	0 = no, 1 = yes


BMI: body mass index. HR: heart rate. CVD: coronary vessel disease. CHF: chronic hepatic failure. CRF: chronic renal failure. AF: atrial fibrillation.

#### Statistical analysis

Baseline data are presented as mean ± standard deviation for continuous variables or frequency (%) for categorical variables. Differences between continuous variables were calculated using rank-sum nonparametric tests for independent samples and categorical variables were compared by χ^2^ tests.

We evaluated the association between sleep duration and hypertension using four multivariate logistic regression models to calculate the odds ratios (OR) and 95% confidence intervals (95% CI). Model 1 included age, educational status, smoking, vegetable consumption, fruit consumption, outdoor activities, diabetes, hyperhomocysteinemia, CVD, CRF, AF, cerebral infarction, cerebral hemorrhage, use of antihypertensives, use of lipid-lowering agents, and blood glucose. Model 2 included model 1 plus BMI. Model 3 included model 2 plus hyperlipidemia. Model 4 included model 3 plus HR. Data analysis and processing were carried out using SPSS26.0 statistical software. A two-sided *P* < 0.05 was considered significant.

## Results

### Baseline characteristics of all participants

The baseline characteristics of all participants are shown in ***Table 2***. A total of 20,053 participants aged 23–98 years were included in this study. The overall incidence of hypertension was 51.2%, including 44.2% men and 55.8% women. Overall, 74% of participants had a normal sleep duration, 19.3% had a short sleep duration, and 6.7% had a long sleep duration. Participants with hypertension were predominantly female, older, and had a higher BMI, faster HR, higher blood sugar, and lower educational status. Analysis of the baseline characteristics of all participants showed that age, educational status, BMI, HR, blood sugar, smoking, outdoor activities, vegetable consumption, fruit consumption, sleep duration, diabetes, hyperlipidemia, hyperhomocysteinemia, CVD, CRF, AF, cerebral infarction, cerebral hemorrhage, use of antihypertensives, use of lipid-lowering agents all differed significantly between the two groups (*P* < 0.05). However, there were no significant differences with regard to sex, drinking, and CHF (*P* > 0.05).

**Table 2:** Baseline characteristics of all participants by hypertension status.


VARIABLE	HYPERTENSION		*P*

	YES	NO	

Sex(%)			0.378

male	4,533(44.2)	4,265(43.6)	

female	5,728(55.8)	5,527(56.4)	

Age	71 ± 8	66 ± 11	<0.01

BMI	25 ± 3	24 ± 3	<0.01

HR	74 ± 11	73 ± 11	<0.01

Blood glucose	6.0 ± 2.0	5.9 ± 1.8	0.002

Educational status(%)			<0.01

primary	7,611(74.2)	6,713(68.6)	

middle	1,817(17.7)	1,915(19.6)	

high	650(6.3)	733(7.5)	

college	182(1.8)	429(4.4)	

master	1(0.0)	2(0.0)	

Smoking(%)			0.007

never	8,020(78.2)	7,531(76.9)	

current	1,425(13.9)	1,464(15.0)	

past	198(1.9)	150(1.5)	

long history of second-hand smoke inhalation	618(6.0)	647(6.6)	

Drinking(%)			0.428

never	8,504(82.9)	8,047(82.2)	

current	1,630(15.9)	1,617(16.5)	

past	127(1.2)	128(1.3)	

Outdoor activities(%)			<0.01

≥20	4,681(45.6)	3,854(39.4)	

5–20	4,379(42.7)	4,560(46.6)	

≤5	1,201(11.7)	1,378(14.1)	

Vegetable consumption(%)			<0.01

≥5	7,433(72.4)	6,681(68.2)	

3–5	2,226(21.7)	2,545(26.0)	

≤3	602(5.9)	566(5.8)	

Fruit consumption(%)			0.017

≥3	4,005(39.0)	3,985(40.7)	

<3	6,256(61.0)	5,807(59.3)	

Sleep duration(%)			<0.01

6–8	7,593(74.0)	7,567(77.3)	

<6	1,982(19.3)	1,534(15.7)	

>8	686(6.7)	691(7.1)	

Diabetes(%)			<0.01

yes	1,894(18.5)	1,230(12.6)	

no	8,367(81.5)	8,562(87.4)	

Hyperlipidemia(%)			<0.01

yes	2,065(20.1)	1,426(14.6)	

no	8,196(79.9)	8,366(85.4)	

Hyperhomocysteinemia(%)			<0.01

yes	52(0.5)	5(0.1)	

no	10,209(99.5)	9,787(99.9)	

CVD(%)			<0.01

yes	604(5.9)	224(2.3)	

no	9,657(94.1)	9,568(97.7)	

CHF(%)			0.093

yes	120(1.2)	141(1.4)	

no	10,141(98.8)	9,651(98.6)	

CRF(%)			0.001

yes	36(0.4)	12(0.1)	

no	10,225(99.6)	9,780(99.9)	

AF(%)			0.008

yes	51(0.5)	26(0.3)	

no	10,210(99.5)	9,766(99.7)	

Cerebral infarction(%)			0.001

yes	173(1.7)	110(1.1)	

no	10,088(98.3)	9,682(98.9)	

Cerebral hemorrhage(%)			<0.01

yes	50(0.5)	11(0.1)	

no	10,211(99.5)	9,781(99.9)	

Use of antihypertensives(%)			<0.01

yes	7,578(73.9)	0(0.0)	

no	2,683(26.1)	9,792(100.0)	

Use of lipid-lowering agents(%)			<0.01

Yes	434(4.2)	182(1.9)	

no	9,827(95.8)	9,610(98.1)	


A *P* < 0.05 was considered significant. BMI: body mass index. HR: heart rate. CVD: coronary vessel disease. CHF: chronic hepatic failure. CRF: chronic renal failure. AF: atrial fibrillation.

### Unadjusted and adjusted analysis of the association between sleep duration and hypertension

There were significant differences among the three sleep-duration groups in terms of hypertension (χ^2^ = 46.2, *P* < 0.01) (***Table 3***). Unadjusted analysis showed that participants with short (<6 h/day) and long (>8 h/day) sleep durations had greater risks of hypertension than those with a normal (6–8 h/day) sleep duration. The risk of hypertension was significantly increased by 30.1% in participants with a long (>8 h/day) sleep duration compared with those with a normal (6–8 h/day) sleep duration (OR = 1.301, *P* < 0.01, 95% CI = 1.149–1.475), while the risk of hypertension was increased by 1.1% higher in participants with a short (<6 h/day) sleep duration compared with those with a normal (6–8 h/day) sleep duration, but the difference was not significant (OR = 1.011, *P* = 0.849, 95% CI = 0.905–1.129).

**Table 3:** Unadjusted analysis between sleep duration and hypertension.


SLEEP DURATION	TOTAL	HYPERTENSION (%)				χ^2^ TEST			LOGISTIC REGRESSION ANALYSIS		
		
		NO	YES			χ^2^	*P*		OR	*P*	95%CI

Normal (6–8h/day)	15,160	7,567 (49.9)	7,593 (50.1)			46.2	<0.01		*		
	
Short (<6h/day)	3,516	1,534 (43.6)	1,982 (56.4)	^#^					1.011	0.849	0.905–1.129
	
Long (>8h/day)	1,377	691 (50.2)	686(49.8)						1.301	<0.010	1.149–1.475


* Compared with normal (6–8 h/day) and long (>8 h/day), it was statistically significant.

**Figure 2:**
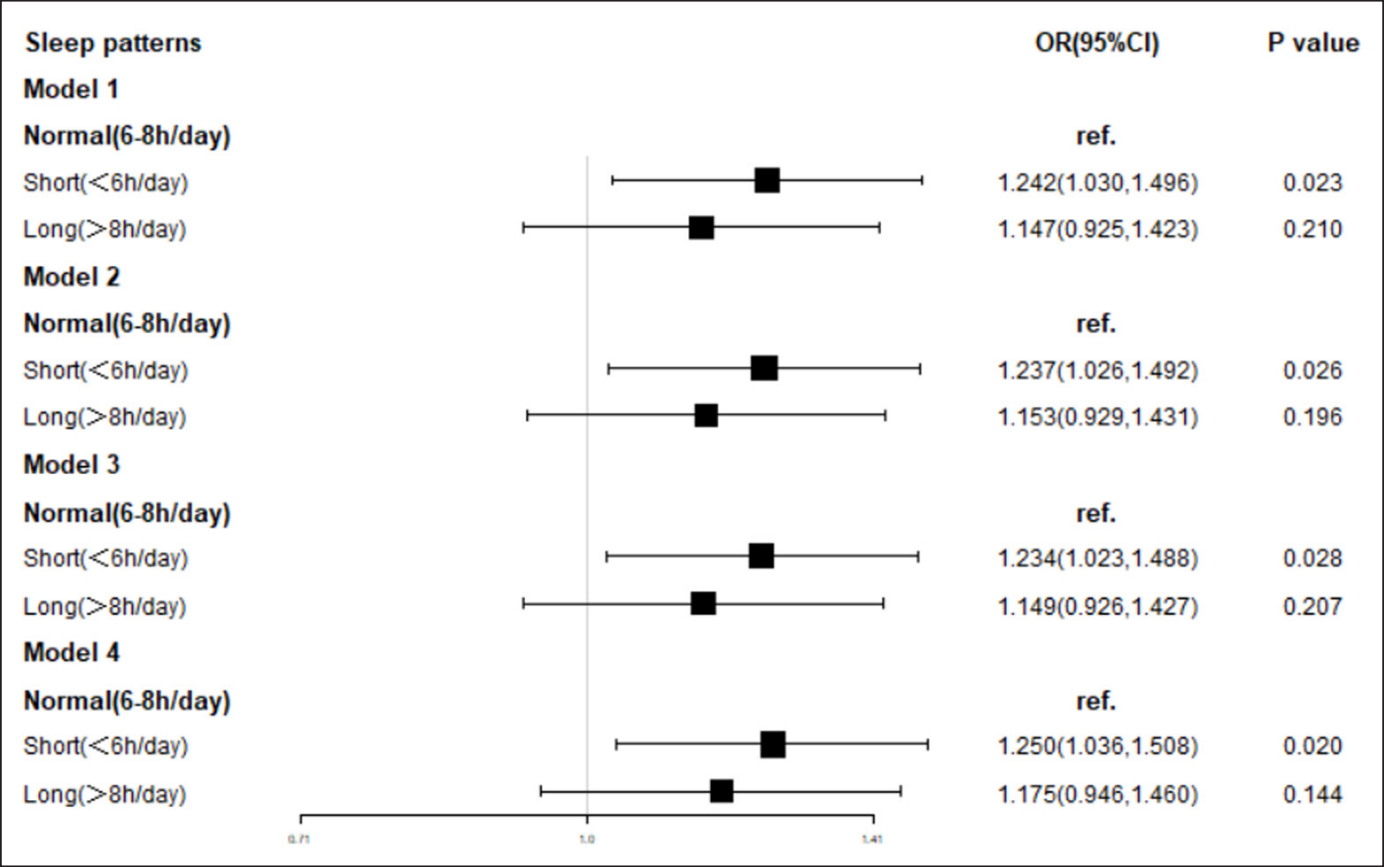
To evaluate the association between sleep duration and hypertension by four multivariate logistic regression models.

***Figure 2*** represented that the risk of hypertension was significantly increased by 24.2% in participants with a short (<6 h/day) sleep duration compared with those with a normal (6–8 h/day) sleep duration after adjusting for age, education status, smoking, vegetable consumption, fruit consumption, outdoor activities, diabetes, hyperhomocysteinemia, CVD, CRF, AF, cerebral infarction, cerebral hemorrhage, use of antihypertensives, use of lipid-lowering agents, and blood sugar (OR = 1.242, *P* < 0.023, 95% CI = 1.030–1.496). In contrast, the increase of 14.7% in participants with a long (>8 h/day) sleep duration was not significant (OR = 1.147, *P* = 0.210, 95% CI = 0.925–1.423). Similar results were obtained after adding BMI **(model 2)**, hyperlipidemia **(model 3)**, or HR **(model 4)** as confounding factors. After fully adjusting for confounding factors (model 4), the risk of hypertension was thus increased significantly by 25% in participants with a short (<6 h/day) sleep duration compared with those with a normal (6–8 h/day) sleep duration (OR = 1.25, *P* = 0.020, 95% CI = 1.036–1.508), while the increase of 17.5% in participants with a long sleep duration was not significant (OR = 1.175, *P* = 0.144, 95% CI = 0.946–1.460).

**Model 1** included age, educational status, smoking, vegetable consumption, fruit consumption, outdoor activities, diabetes, hyperhomocysteinemia, CVD, CRF, AF, cerebral infarction, cerebral hemorrhage, use of antihypertensives, use of lipid-lowering agents, and blood glucose. **Model 2** included **model 1** plus BMI. **Model 3** included **model 2** plus hyperlipidemia. **Model 4** included **model 3** plus HR. The odds ratios (OR) and 95% confidence interval (95% CI) were presented in ***Figure 2***.

## Discussion

Given the potential association between sleep duration and hypertension, this study evaluated the association between self-reported sleep duration and hypertension among adults in southwestern China. Multivariate logistic regression analysis demonstrated that the risk of hypertension was increased in participants with either a short (<6 h/day) or a long (>8 h/day) sleep duration compared with participants with a normal (6–8 h/day) sleep duration. **Model 1** showed that a short (<6 h/day) sleep duration significantly increased the risk of hypertension by 24.2% compared with participants with a normal (6–8 h/day) sleep duration (OR = 1.242, *P* < 0.023, 95% CI = 1.030–1.496), while a long (>8 h/day) sleep duration increased the risk by 14.7%, which was not significant (OR = 1.147, *P* = 0.21, 95% CI = 0.925–1.423). These differences remained after controlling for BMI **(model 2)**, hyperlipidemia **(model 3)**, and HR **(model 4)**. These results indicated that participants with a short (<6 h/day) sleep duration were much more prone to develop hypertension, suggesting that sleep had a protective effect against hypertension among adults in southwestern China.

Our study concluded that insufficient sleep was associated with an increased risk of hypertension, which was consistent with the results of most previous studies [1,2,3,6,7,8,12,13,14,15]. However, our results demonstrated that participants with only a short (<6 h/day) sleep duration had a greater risk of hypertension, while a long (>8 h/day) sleep duration did not significantly increase the risk of hypertension.

Hypertension is a common chronic disease that represents a significant risk factor for cardiovascular disease worldwide. Cardiologists have recently become concerned about the association between sleep duration and hypertension. Several studies have demonstrated the biological mechanism underlying the association between sleep duration and hypertension, and have proposed various possible mechanisms, including the effects of insufficient sleep on biological rhythms [10,16] and hemodynamic changes [9], which are due to increased sympathetic nerve activity, the hypothalamic-pituitary axis, and the oxidative stress response. However, the exact biological mechanism responsible for the association between sleep duration and hypertension remains unclear. Further fundamental and prospective studies are therefore needed to clarify the biological mechanisms and their relationship.

Compared with previous studies, the current study had several strengths. First, the sample was from southwest China and the sample size was large, making it representative of this region. Second, we analyzed the relationship between sleep duration and hypertension by multivariate logistic regression analysis and controlled for a large number of confounding factors. Third, the results thus provide a basis for future studies on the association between sleep duration and hypertension. However, the study also had some limitations. First, sleep duration was based on self-reported data and was thus very subjective, potentially leading to errors in the grouping of some participants. Second, sleep duration did not include the duration of daytime sleep or the time of naps, and the effect of daytime sleep or the time of naps on hypertension was thus unclear. Third, sleep quality was not included in this study, and the effect of sleep quality on hypertension was thus unknown. Forth, this was a cross-sectional study, which reduced the power of the results. Therefore further research is needed using more accurate measurements and evaluation methods for collecting sleep-duration information. More prospective studies are needed to determine the association between sleep duration and hypertension.

## Conclusion

The results of this study suggest that a short (<6 h/day) sleep duration is related to an increased risk of hypertension and is an independent risk factor for hypertension among adults in southwest China. Individuals with a short (<6 h/day) sleep duration are thus more likely to have hypertension, indicating that sleep may help to protect against the development of hypertension. The result of this study has some degree of guiding importance in preventing hypertension in clinical practice.
